# Effectiveness and Safety of Recombinant Human Follicle-Stimulating Hormone (Follitrope™) in Inducing Controlled Ovarian Stimulation in Infertile Women in Real-World Practice: a Prospective Cohort Study

**DOI:** 10.1007/s43032-023-01228-6

**Published:** 2023-04-17

**Authors:** Chang-Woo Choo, Young Sun Ahn, Kyu Hyun Kim, Jae Ho Lee, Kyoung Yong Moon, Bum-Chae Choi, Young Je Kang, Hye Young Kim, Chae Hee Sim, Ji Eun Han, Chung-Hoon Kim, Eun Jeong Jang, Yoojin Lee, Jei Won Moon, Dong Soo Park, Hyung Jae Won, An Na Kim, Ju-Young Kim, Kwang Rye Kim, Ji Hyun Ahn, Joong-Yeup Lee, Heemin Gwak, Ji Hyang Kim

**Affiliations:** 1 Department of Gynecologic Endocrinology, Female Infertility, Fertility Preservation, Seoul Maria Fertility Hospital, Seoul, Republic of Korea; 2 Seoul Women’s Hospital, Incheon, Republic of Korea; 3 Bucheon Maria Fertility Hospital, Bucheon, Republic of Korea; 4 Ilsan Maria Fertility Hospital, Goyang, Republic of Korea; 5 Busan Maria Fertility Hospital, Busan, Republic of Korea; 6 Center for Infertility & Recurrent Miscarriage, Creation & Love Women’s Hospital, Gwangju, Republic of Korea; 7 Pyeongchon Maria Fertility Hospital, Anyang, Republic of Korea; 8 Department of Obstetrics and Gynecology, Fertility Center, Agaon Fertility Clinic, Seoul, Republic of Korea; 9 Mamapapa and Baby Ob/Gy Clinic, Ulsan, Republic of Korea; 10 Miraeyeon Fertility Clinic, Seoul, Republic of Korea; 11 M Fertility Center, Seoul, Republic of Korea; 12 MizMedi Hospital, Seoul, Republic of Korea; 13 CHA Daegu Medical Center, CHA University School of Medicine, Daegu, Republic of Korea; 14 Sarang I IVF Center, Seoul, Republic of Korea; 15 Sae Ran Women’s Clinic, Jeonju, Republic of Korea; 16 Hamchoon Women’s Clinic, Seoul, Republic of Korea; 17 Life Sciences, LG Chem, Ltd., Seoul, Republic of Korea; 18 Department of Obstetrics and Gynecology, Fertility Center of CHA Bundang Medical Center, CHA University School of Medicine, 64 Yatap-ro, Bundang-gu, Seongnam, Gyeonggi-do 13520 Republic of Korea

**Keywords:** Controlled ovarian stimulation, In vitro fertilization, Ovarian response, Pregnancy, Recombinant human follicle-stimulating hormone

## Abstract

To evaluate the safety and effectiveness of recombinant human follicle-stimulating hormone (rhFSH [Follitrope™]) in infertile women undergoing in vitro fertilization (IVF). To identify predictors of ovarian response that induce optimal clinical outcomes. This multicenter prospective study enrolled infertile women who were scheduled to undergo IVF after ovarian stimulation with rhFSH (Follitrope™) following the gonadotropin-releasing hormone (GnRH) agonist or GnRH antagonist protocol. Predictive factors for ovarian response were identified in the GnRH antagonist group based on the number of oocytes retrieved. A total of 516 infertile women were enrolled, among whom 136 (except one who withdrew before administration) received rhFSH using the GnRH agonist protocol and 379 using the antagonist protocol. The mean number of oocytes retrieved was 13.4 in the GnRH agonist group and 13.6 in the GnRH antagonist group. The clinical pregnancy rates were 32.3% (30/93) and 39.9% (115/288) in the GnRH agonist and antagonist groups, respectively. The incidence of ovarian hyperstimulation syndrome was 1.8% and 3.4% in the GnRH agonist and antagonist groups, respectively. No other significant safety risks associated with rhFSH administration were identified. Body mass index, basal serum FSH and anti-Müllerian hormone levels, and antral follicle count were identified as predictors of ovarian response by multiple regression with backward elimination, and the final regression model accounted for 26.5% of the response variability. In real-world practice, rhFSH (Follitrope™) is safe and effective in inducing ovarian stimulation in infertile women. Patient characteristics identified as predictors can be considered to be highly related to optimal clinical outcomes.

**Supplementary Information:**

The online version contains supplementary material available at 10.1007/s43032-023-01228-6.

## Introduction

Among the assisted reproductive techniques used for the treatment of infertility, in vitro fertilization (IVF) and intracytoplasmic sperm injection (ICSI) are the most commonly used [1, 2]. Controlled ovarian hyperstimulation is a key factor in IVF/ICSI, and recombinant human follicle-stimulating hormone (rhFSH), which is produced as a highly purified FSH preparation and has a greater availability and decreased variability, is mainly used for this purpose [3–6].

Pregnancy and live birth rates are influenced by the number of oocytes retrieved [2, 7]. A small number of retrieved oocytes may increase the risk of poor response, which may increase cycle cancellation rates and lower pregnancy rates. However, a large number of retrieved oocytes may increase the risk of inducing a hyperresponse, such as ovarian hyperstimulation syndrome (OHSS) [2, 7, 8]. Therefore, obtaining the optimal number of oocytes needed is important. The administered dose of rhFSH is related to the number of oocytes retrieved, which is a prognostic factor in infertile female patients undergoing IVF [7, 9]. It is also essential to determine the factors predicting the ovarian response to rhFSH in order to induce optimal oocyte production and optimal clinical outcomes (increased clinical efficacy) while minimizing the risk of OHSS.

Follitrope™, developed by LG Chem, Ltd. (Seoul, Republic of Korea) and approved by the Korean Ministry of Food and Drug Safety in 2007, is an rhFSH composed of follitropin alpha and beta subunits [1]. Its clinical efficacy and safety have been confirmed in a randomized, multicenter, assessor-blind, active-controlled phase 3 study [1], and it is now commercially available in several countries. In the above phase 3 clinical trial, the authors evaluated the efficacy and safety of rhFSH (Follitrope™) used with a gonadotropin-releasing hormone (GnRH) agonist protocol. The GnRH antagonist and GnRH agonist protocols are widely used for IVF, with each protocol having advantages and disadvantages based on its own characteristics [10, 11]. The use of the GnRH antagonist or agonist is determined based on the clinician’s discretion with consideration of the patient’s characteristics. However, clinical data assessing the efficacy and safety of rhFSH (Follitrope™) using the GnRH antagonist protocol are still lacking, and there is a need for the evaluation of the clinical outcomes of rhFSH (Follitrope™) using this protocol. Therefore, this observational study on the use of Follitrope™ was conducted to evaluate the safety and effectiveness of rhFSH in infertile women undergoing IVF/ICSI in real-world practice using both the GnRH agonist and antagonist protocols. Along with the evaluation of the effectiveness and safety of Follitrope™, predictive factors of ovarian response that induce optimal clinical outcomes in IVF when using Follitrope™ in real-world practice were also identified.

## Materials and Methods

### Study Design

This multicenter prospective cohort study was conducted from August 2018 to July 2020 at 18 study centers in the Republic of Korea. The study was conducted in compliance with the principles of the Declaration of Helsinki and local regulatory guidelines and was approved by the institutional review board in accordance with the standards of the Korean regulatory authority. Written informed consent was obtained from all participants, and the study was registered at ClinicalTrials.gov (NCT04227171).

### Participants

Infertile women aged 19–39 years with a menstrual cycle of 25–35 days who were scheduled to undergo IVF after stimulation with rhFSH (Follitrope™, LG Chem, Ltd., Seoul, Republic of Korea) using the GnRH agonist or GnRH antagonist protocol were included in the study. The main exclusion criteria were as follows: (1) three or more previous IVF cycles, (2) a medical history of polycystic ovary syndrome, and (3) a poor ovarian response, according to the Bologna criteria [12]. The criteria for abnormal ovarian reserve in the poor ovarian responder criteria were determined at the discretion of the investigator (antral follicle count [AFC] < 5–7 follicles, anti-Müllerian hormone [AMH] < 0.5–1.1 ng/mL). Patients with contraindications to rhFSH (Follitrope™) were also excluded.

### Study Procedures

**Fig. 1:**
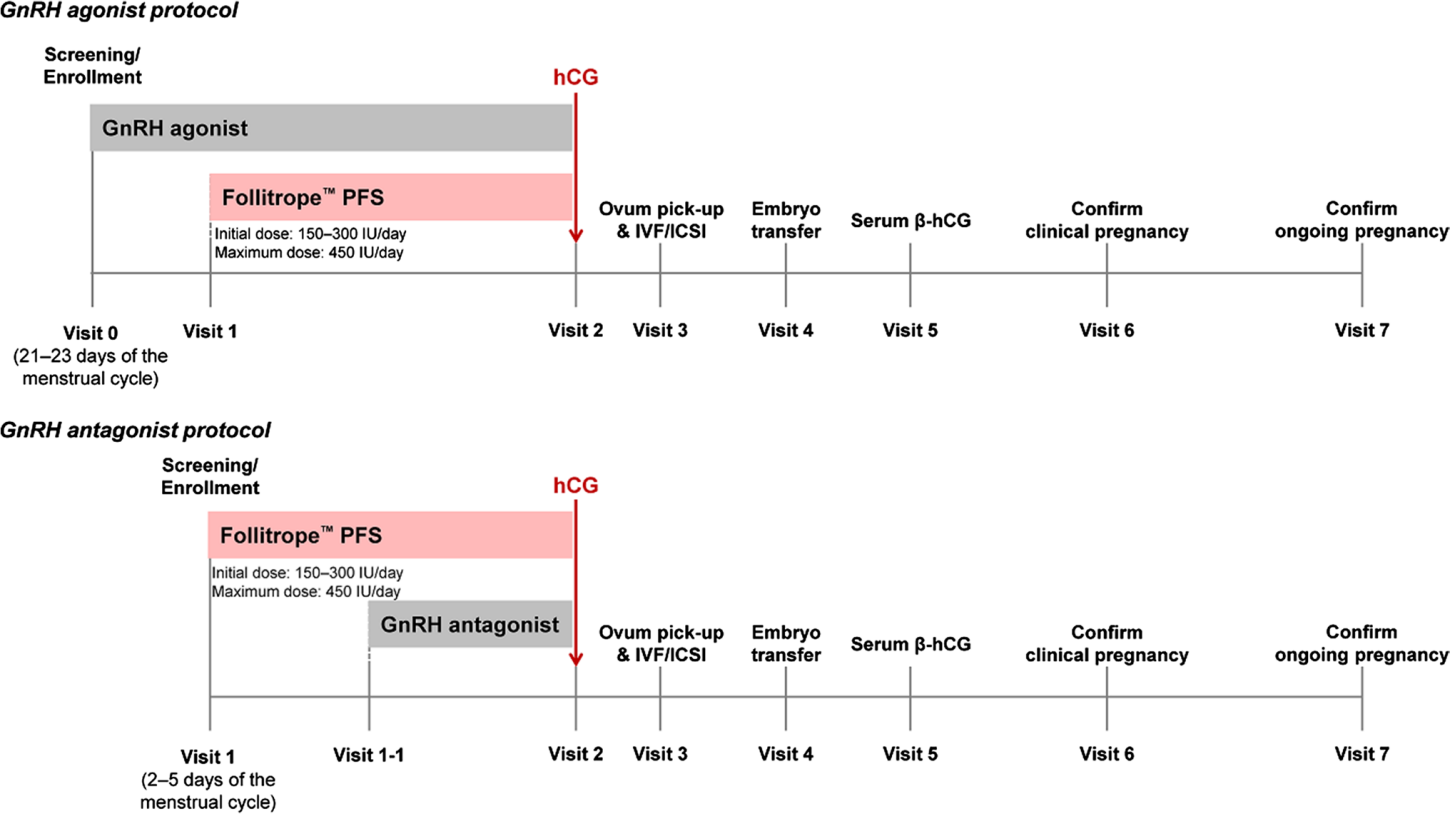
Study design diagram. GnRH, gonadotropin-releasing hormone; hCG, human chorionic gonadotropin; ICSI, intracytoplasmic sperm injection; IVF, in vitro fertilization; PFS, prefilled syringe

In the patients enrolled in this study, controlled ovarian stimulation was induced according to the GnRH agonist or GnRH antagonist protocol. The choice of the protocol to use (GnRH agonist or GnRH antagonist protocol) in each patient was determined by the investigator prior to the patient’s enrollment and was based on the patient’s specific characteristics and the investigator’s discretion. The design and procedures of this clinical study are presented in Fig. 1.

Patients undergoing IVF using the GnRH agonist protocol were enrolled in this study on days 21–23 of their menstrual cycles. Starting on the day of enrollment, they were administered GnRH agonists until the next menstrual cycle started and pituitary downregulation was reached (e.g. estradiol (E_2_) level ≤ 50 pg/mL). The GnRH agonist (leuprorelin, triptorelin, or goserelin) and its dose were determined at the discretion of the investigator. At this time, the administration of rhFSH (Follitrope™) was initiated. The administration dose of rhFSH was generally 150–300 IU, and it was administered subcutaneously until adequate follicle development was reached. Its dose was adjusted throughout depending on the ovarian response (E_2_ level, follicle size, follicle number, etc.), and no more than 450 IU of rhFSH were administered daily.

The administrations of GnRH agonist and rhFSH were stopped (at the discretion of the investigator) when follicle development was reached; for example, when three or more follicles with a diameter of 17 mm or more were observed. Human chorionic gonadotropin (hCG) was administered once within 48 h from the last rhFSH administration to induce ovulation. For the hCG, choriogonadotropin alpha (Ovidrel [Merck KGaA, Darmstadt, Germany] 250 μg or 500 μg) or chorionic gonadotropin (IVF-C [LG Chem, Ltd., Seoul, Republic of Korea] or Pregnyl [MSD, Kenilworth, NJ, USA] 5000 IU or 10,000 IU) were used.

Approximately 36 h after the hCG trigger, oocytes were retrieved, and fertilization was performed using IVF, ICSI, or IVF+ICSI (at the discretion of the investigator). After fertilization, one to three embryos were transferred to each subject, and the number of embryos transferred was determined at the physician’s discretion considering the patient’s age, embryo quality, and the number of previous cycles. For luteal support, progesterone was administered for up to 12 weeks after oocyte retrieval, at the investigator’s discretion. Progesterone or hydroxyprogesterone was administered mainly intravaginally or by intramuscular injection. Serum β-hCG concentration was measured to confirm biochemical pregnancy approximately 14 days after the date of oocyte retrieval. Clinical pregnancy was confirmed through ultrasound approximately 24 days after oocyte retrieval, and ongoing pregnancy was confirmed at least 10 weeks after ovum pick-up.

For the enrolled patients who underwent IVF using the GnRH antagonist protocol, rhFSH (Follitrope™) administration was started on days 2–5 of the menstrual cycle. The administration dose of rhFSH was generally 150–300 IU, as in patients undergoing IVF using the GnRH agonist protocol. The rhFSH was administered until adequate follicle development was reached while adjusting its dose based on ovarian response. The maximum daily dose of rhFSH was 450 IU. On days 6–8 of the menstrual cycle, if the ovarian response was adequate (e.g. E_2_ level > 200 pg/mL, leading follicle size ≥ 12 mm), administration of the GnRH antagonist was initiated. The choice of the GnRH antagonist (ganirelix 0.25 mg or cetrorelix 0.25 mg) was determined at the discretion of the investigator.

Administration of the GnRH antagonist and rhFSH was stopped (at the discretion of the investigator) when follicle development was reached, and hCG was administered once within 48 h after the last rhFSH administration to induce ovulation. The subsequent procedures were the same as those for the subjects undergoing IVF using the GnRH agonist protocol.

The total study period per subject could vary depending on the GnRH agonist or GnRH antagonist protocol and on whether or not the patient got pregnant; it was up to approximately 14 weeks. Ultrasound examination and hormone assays were performed at the discretion of the investigator during the study period. Serum FSH, luteinizing hormone (LH), E_2_, progesterone (P_4_), and AMH levels were measured at each study center using a standardized method.

### Study Outcomes

The study outcomes used to evaluate the effectiveness of rhFSH (Follitrope™) included the following: the number of retrieved oocytes, the total dose and duration of rhFSH administration, serum E_2_ and P_4_ concentrations on the day of hCG administration, the number of follicles with a diameter of 14 mm or more on the day of hCG administration, oocyte maturity quality (proportion of metaphase II oocytes, for ICSI cases only), fertilization rate, number of embryos transferred, implantation rate, biochemical pregnancy rate (β-hCG positive rate), clinical pregnancy rate, and ongoing pregnancy rate. The metaphase II oocyte rate (proportion) was calculated as the number of metaphase II oocytes divided by the number of oocytes for which ICSI was performed. The fertilization rate was calculated as the number of two pronuclei (PN) oocytes divided by the number of oocytes used for fertilization. The implantation rate was defined as the total number of gestational sacs divided by the total number of embryos transferred to all the subjects. Biochemical pregnancy was defined as a positive serum β-hCG. Clinical pregnancy was defined as the presence of a gestational sac approximately 24 days after ovum pick-up. Ongoing pregnancy was defined as the presence of gestational sacs and a fetal heartbeat at least 10 weeks after ovum pick-up.

The safety of rhFSH (Follitrope™) was evaluated by monitoring adverse events including OHSS.

### Statistical Analysis

Statistical hypotheses and tests were not established as this study aimed to evaluate the effectiveness and safety of rhFSH (Follitrope™) in an exploratory manner in real-world clinical practice. A sample size of at least 400 and up to 1000 was calculated as the number of subjects that could be recruited for approximately 1 year after the start of this study. Regarding safety evaluation, if 1000 subjects were given the study drug and an adverse event of special interest was not observed, it could be concluded with a 95% probability that the true incidence of adverse events was less than 0.3% (3/1000, rule of three). Similarly, the minimum number of 400 subjects was the sample size, providing a 95% probability of observing at least one adverse event with a true incidence rate of 0.75%.

Enrolled subjects who were administered rhFSH at least once were included in the safety set. Among those, subjects for whom at least one effectiveness endpoint was measured after rhFSH administration were included in the effectiveness set.

All data were analyzed using the software SAS® version 9.4 (SAS Institute, Inc., Cary, NC, USA) and presented for the GnRH agonist and GnRH antagonist groups. For the GnRH agonist group, the data of subjects using the GnRH agonist long protocol, commonly used among GnRH agonist protocols for IVF, were analyzed and presented. Demographic and baseline characteristics, effectiveness, and safety data were summarized as descriptive statistics. A comparative test between the GnRH agonist and GnRH antagonist groups was performed using Student’s *t*-test, chi-square, or Fisher’s exact tests as appropriate for the exploratory purpose. *P*-values < 0.05 were considered to be statistically significant. No imputation was performed for missing or incomplete data.

Predictive factors of ovarian response were identified in the GnRH antagonist group using multiple regression analysis with backward elimination based on the number of oocytes retrieved. The evaluated predictive factors were age, smoking status, body weight, body mass index (BMI), mean menstrual cycle, number of previous IVF cycles, basal serum hormones (FSH, LH, E_2_, and AMH) levels, and AFC. The basal serum P_4_ levels were excluded from the model because of the small quantity of data collected. The cause of infertility was also excluded from the model because it was not clinically relevant in predicting ovarian response. The random forest method was also used to identify the predictors. Variables that reached statistical significance (*P*-values < 0.1) in the multiple regression with backward elimination were included in the final model for predicting ovarian response.

## Results

### Subjects’ Dispositions and Baseline Characteristics

**Fig. 2:**
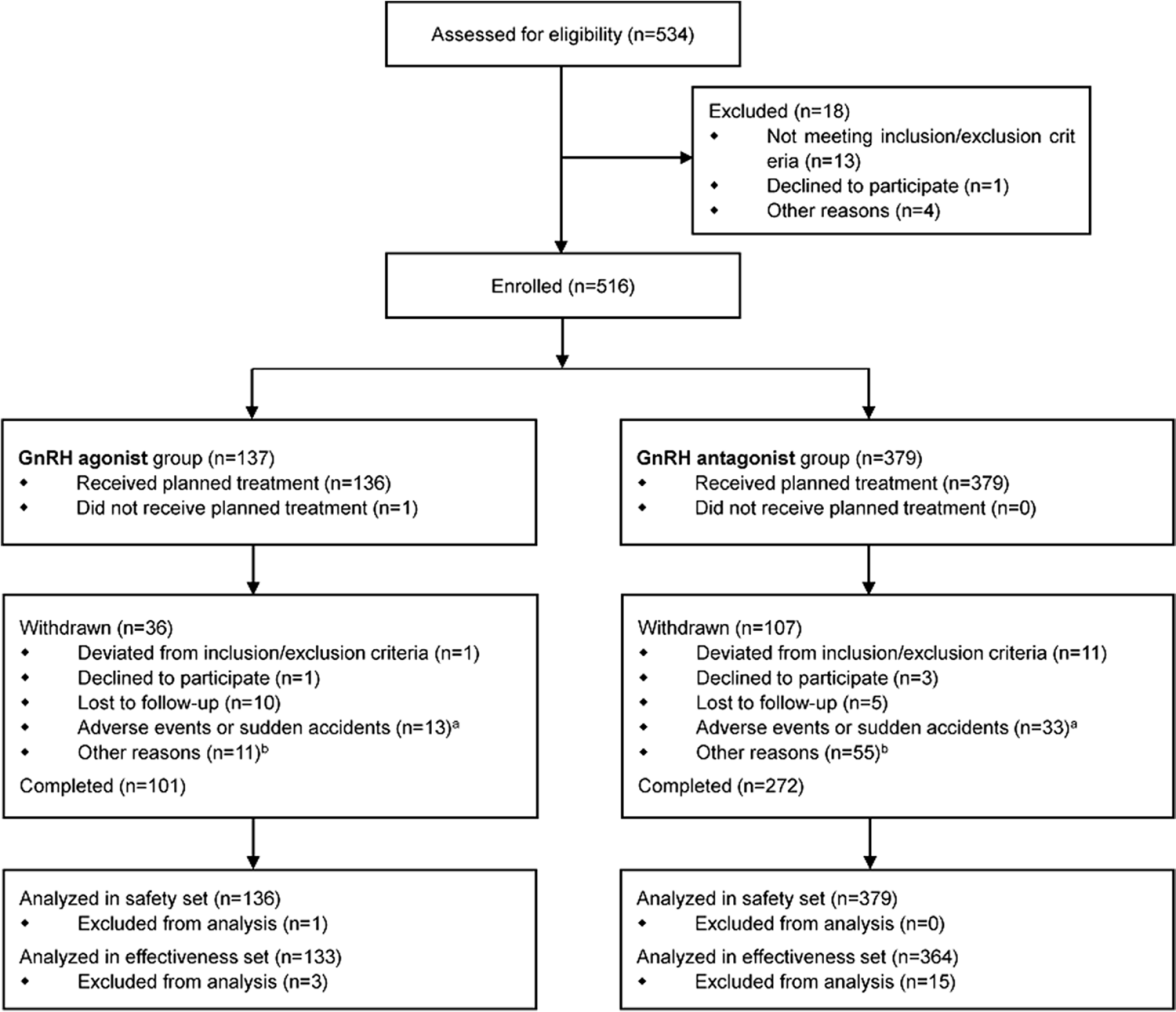
Subject disposition. Superscript letter a, the most common adverse events leading to withdrawal were increase in progesterone level and ectopic pregnancy. Superscript letter b. among the other reasons for withdrawal, embryo transfer failure and embryo cryopreservation due to increased progesterone levels were the most common. GnRH, gonadotropin-releasing hormone

A total of 534 infertile female patients were screened, and 516 were enrolled in this study after excluding those who were not eligible for this study (according to the inclusion/exclusion criteria) and those who withdrew their consent (Fig. 2). Among the enrolled patients, 136 (excluding one subject who dropped out prior to rhFSH administration) received rhFSH using the GnRH agonist protocol (GnRH agonist group) and 379 received rhFSH using the GnRH antagonist protocol (GnRH antagonist group). Among the subjects enrolled in the GnRH agonist group, 112 (82.4%) were administered rhFSH using the agonist long protocol, and 24 (17.6%) underwent the short protocol. A total of 515 subjects who received rhFSH at least once were included in the safety set, among whom 133 in the GnRH agonist group and 364 in the GnRH antagonist group were included in the effectiveness set (Fig. 2). For the GnRH agonist group, the data of subjects using the long protocol were included in the final analysis (112 subjects analyzed for safety and effectiveness).

The mean (± standard deviation [SD]) age of subjects was 34.5 (± 2.8) years in the GnRH agonist group and 33.9 (± 3.1) years in the GnRH antagonist group, and the mean (± SD) duration of infertility was 2.6 (± 1.5) years in the GnRH agonist group and 2.4 (± 1.6) years in the GnRH antagonist group. A total of 87.3% of the participants had no experience with IVF procedures before participating in this study. The baseline characteristics are presented in Table 1, showing no significant difference between the GnRH agonist and GnRH antagonist groups in general. However, BMI, basal serum FSH, and AMH level showed a significant difference. The initial and total doses of rFSH were higher in the GnRH agonist group than in the GnRH antagonist group (251.1 vs. 220.9 IU and 2395.3 vs. 2069.5 IU, respectively) (Table 2).

**Table 1:** Baseline characteristics (effectiveness set)

	GnRH agonist (*N* = 112)	GnRH antagonist (*N* = 364)	*P*-value
Age (years)	34.5 ± 2.8	33.9 ± 3.1	0.104
BMI (kg/m^2^)	21.5 ± 3.2	22.3 ± 3.5	0.031
Smoking status, *n* (%)			0.363
Yes	3 (2.7%)	4 (1.1%)	
No	109 (97.3%)	360 (98.9%)	
Duration of menstrual cycle (days)	29.1 ± 2.1	29.5 ± 2.1	0.114
Duration of infertility (years)	2.6 ± 1.5	2.4 ± 1.6	0.166
Cause of infertility^a^, *n* (%)			0.919
Male factor	50 (44.6%)	150 (41.2%)	
Unknown/Unexplained	49 (43.8%)	155 (42.6%)	
Tubal factor	22 (19.6%)	81 (22.3%)	
Uterine factor	6 (5.4%)	19 (5.2%)	
Number of previous IVF cycles, *n* (%)			0.547
None	99 (88.4%)	315 (86.5%)	
1	13 (11.6%)	43 (11.8%)	
2	0 (0.0%)	6 (1.6%)	
Serum hormone levels at screening			
FSH (mIU/mL)	6.6 ± 2.6	7.5 ± 2.4	0.015
LH (mIU/mL)	5.9 ± 2.9	6.1 ± 3.7	0.601
E_2_ (pg/mL)	122.9 ± 402.5	88.3 ± 372.6	0.529
AMH (ng/mL)	3.0 ± 1.8	3.7 ± 2.2	0.001
AFC at visit 1 (prior to rhFSH administration)	10.6 ± 4.9	11.5 ± 5.7	0.203
Fertilization procedure, *n* (%)			0.562
IVF	54 (49.1%)	179 (49.4%)	
ICSI	32 (29.1%)	119 (32.9%)	
Both IVF/ICSI	24 (21.8%)	64 (17.7%)	

Data are presented as mean ± standard deviation for continuous variables and as the number and percentage of subjects for categorical variables unless otherwise indicated

*AFC*, antral follicle count; *AMH*, anti-Müllerian hormone; *BMI*, body mass index; *E*_2_, estradiol; *FSH*, follicle-stimulating hormone; *GnRH*, gonadotropin-releasing hormone; *ICSI*, intracytoplasmic sperm injection; *IVF*, in vitro fertilization; *LH*, luteinizing hormone; *rhFSH*, recombinant human FSH

^a^Causes of infertility were counted as duplicates (if multiple factors were the cause, each was counted)

**Table 2:** Clinical outcomes (effectiveness set)

	GnRH agonist (*N* = 112)	GnRH antagonist (*N* = 364)	*P*-value
Number of retrieved oocytes	13.4 ± 6.4	13.6 ± 7.5	0.791
Initial dose of rhFSH (IU)	251.1 ± 55.8	220.9 ± 54.4	< 0.001
Duration of stimulations with rhFSH (days)	9.6 ± 1.5	9.4 ± 1.9	0.147
Total dose of rhFSH administered (IU)	2395.3 ± 644.9	2069.5 ± 597.9	< 0.001
Serum E_2_ level on hCG trigger day (pg/mL)	3169.4 ± 1941.1	2976.7 ± 2242.9	0.515
Serum P_4_ level on hCG trigger day (ng/mL)	0.9 ± 0.6	1.1 ± 1.3	0.151
Number of follicles with a diameter of ≥ 14 mm on hCG trigger day	11.3 ± 4.0	10.3 ± 4.6	0.098
Metaphase II oocyte rate: ICSI case only (%)	74.1 ± 34.1	77.1 ± 29.4	0.516
Fertilization rate (%)	77.1 ± 15.6	75.7 ± 20.1	0.451
Number of embryos transferred	1.8 ± 0.5	2.0 ± 0.6	0.066
Implantation rate (%)	20.3 (35/172)	25.8 (147/569)	0.143
Biochemical pregnancy rate (%)	34.4 (32/93)	45.1 (130/288)	0.069
Clinical pregnancy rate (%)	32.3 (30/93)	39.9 (115/288)	0.185
Clinical pregnancy rate per oocyte pick-up (%)	26.8 (30/112)	31.7 (115/363)	0.325
Clinical pregnancy rate with fetal heartbeat (%)	23.7 (22/93)	35.8 (103/288)	0.031
Ongoing pregnancy rate (%)	18.3 (17/93)	28.1 (81/288)	0.059

Data are presented as mean ± standard deviation for continuous variables and as the number and percentage of subjects for categorical variables unless otherwise indicated

*E*_2_, estradiol; *GnRH*, gonadotropin-releasing hormone; *hCG*, human chorionic gonadotropin; *ICSI*, intracytoplasmic sperm injection; *P*_4_, progesterone; *rhFSH*, recombinant human follicle-stimulating hormone

### Clinical Outcomes

The mean (± SD) number of oocytes retrieved was 13.4 (± 6.4) in the GnRH agonist group and 13.6 (± 7.5) in the GnRH antagonist group (Table 2). Among the subjects included in the effectiveness set, 93 and 288 in the GnRH agonist and GnRH antagonist groups, respectively, underwent embryo transfer. A total of 58 subjects (eight in the GnRH agonist group and 50 in the GnRH antagonist group) did not undergo embryo transfer, and there were 37 subjects (11 in the GnRH agonist group and 26 in the GnRH antagonist group) for whom we could not determine (due to reasons such as dropout from the study prior to the embryo transfer procedure) whether or not they underwent embryo transfer.

**Fig. 3:**
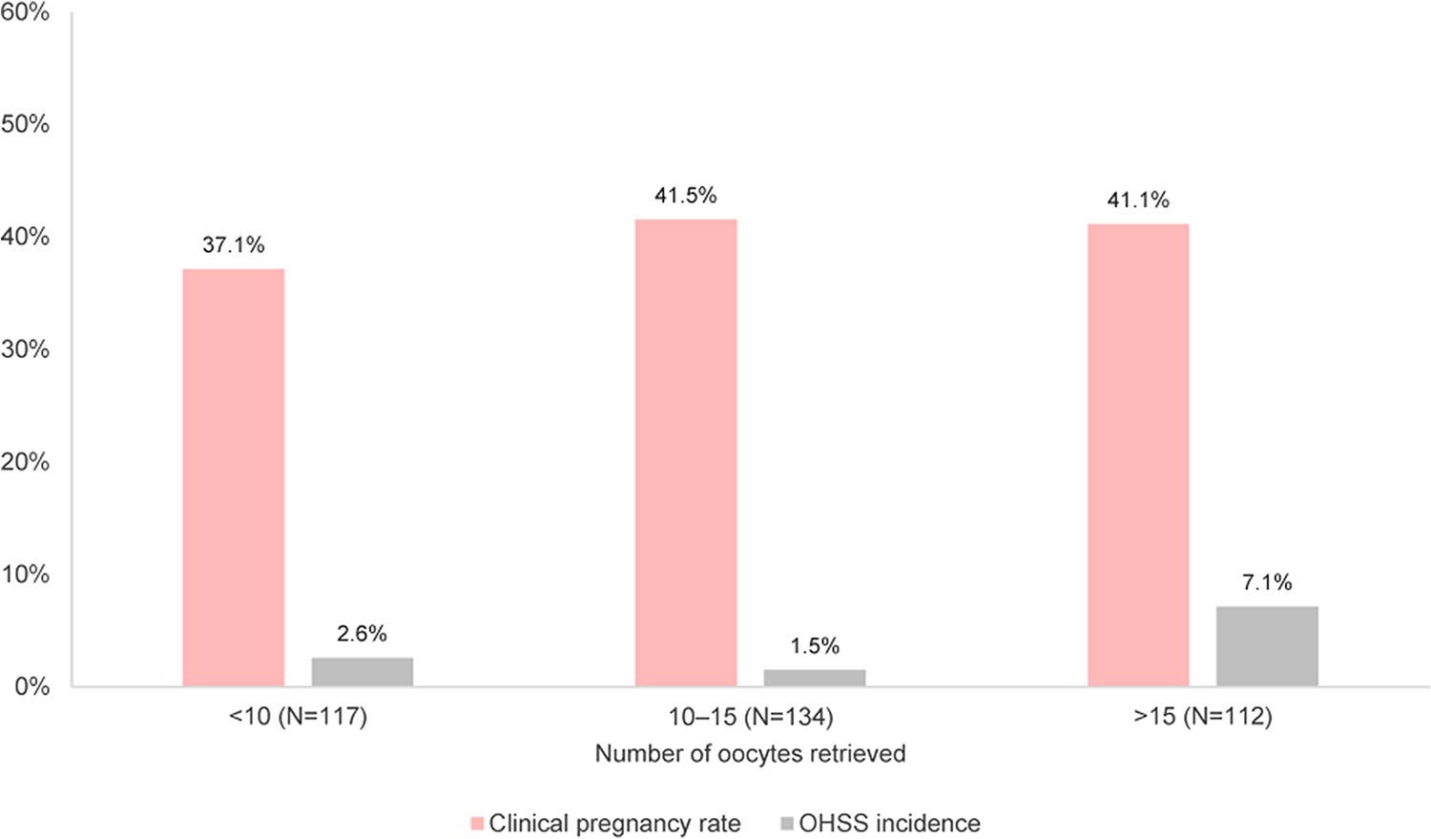
Clinical outcomes according to the number of retrieved oocytes in the GnRH antagonist group (effectiveness set). The difference in clinical pregnancy rates among the three subgroups was not significant according to Pearson’s chi-square test (*P*-value = 0.784). However, the incidence of OHSS was significantly different among the three subgroups according to Fisher’s exact test (*P*-value = 0.047). GnRH, gonadotropin-releasing hormone; OHSS; ovarian hyperstimulation syndrome

The biochemical pregnancy rate, calculated as the percentage of subjects with a positive serum β-hCG test result, among the subjects who underwent embryo transfer was 34.4% (32/93) in the GnRH agonist group and 45.1% (130/288) in the GnRH antagonist group. The clinical pregnancy rates were 32.3% (30/93) in the GnRH agonist group and 39.9% (115/288) in the GnRH antagonist group. There was no difference observed in the biochemical pregnancy rate and the clinical pregnancy rate between the two groups. Although the clinical pregnancy rate with a fetal heartbeat was significantly higher in the GnRH antagonist group, there was no significant difference in the ongoing pregnancy rate confirmed at least 10 weeks after ovum pick-up between the two groups. As for the subgroup results based on the number of oocytes retrieved, the clinical pregnancy rate in the subgroup with 10–15 retrieved oocytes was slightly higher than that in the other two subgroups (< 10 and > 15, Fig. 3 and Supplementary Table S1). Additionally, subgroup analysis was performed based on the agent of GnRH antagonist used; the results are presented in Supplementary Table S2. The overall clinical outcomes were similar between the subgroups using ganirelix and cetrorelix as GnRH antagonists, and the ongoing pregnancy rate was significantly higher in the subgroup using ganirelix than that in the subgroup using cetrorelix.

The incidence of adverse events was 25.9% and 35.6% in the GnRH agonist and antagonist groups, respectively (Table 3). The most common adverse events were vaginal hemorrhage and increased progesterone (Table 3). Most adverse events were mild in severity; no severe adverse events were reported in the GnRH agonist and antagonist groups. There was no significant difference in the incidence of OHSS between the GnRH agonist and antagonist groups (1.8% vs. 3.4%). OHSS was reported as a serious adverse event requiring hospitalization in three subjects (0.8%) in the GnRH antagonist group. The severity of OHSS was mostly mild, and most of the subjects recovered during the study period. Regarding the subgroups based on the number of retrieved oocytes, the incidence of OHSS was significantly higher in the subgroup with more than 15 oocytes retrieved than in the subgroup with 15 oocytes or less (7.1% vs. 2.0%, *P =* 0.027). However, the incidence of OHSS was comparable in the subgroups with “10 to 15” and “less than 10” retrieved oocytes (1.5% vs. 2.6%, Fig. 3). There was a significant difference in the incidence of OHSS among the three subgroups (*P*-value = 0.047, Fig. 3). None of the subjects discontinued the study drug due to adverse events, and no other notable safety risks associated with rhFSH were observed.

**Table 3:** Safety results (Safety set)

	GnRH agonist (*N* = 112)	GnRH antagonist (*N* = 379)	*P*-value
Any adverse events, *n* (%)	29 (25.9%)	135 (35.6%)	0.055
Common adverse events, *n* (%)			
Vaginal haemorrhage	8 (7.1%)	27 (7.1%)	0.995
Ovarian hyperstimulation syndrome	2 (1.8%)	13 (3.4%)	0.538
Mild	1 (0.9%)	10 (2.6%)	
Moderate	1 (0.9%)	3 (0.8%)	
Severe	0 (0.0%)	0 (0.0%)	
Ectopic pregnancy	1 (0.9%)	13 (3.4%)	0.207
Any adverse drug reactions, *n* (%)	1 (0.9%)	22 (5.8%)	0.031
Any serious adverse events, *n* (%)	0 (0.0%)	7 (1.8%)	0.360
Any serious adverse drug reactions, *n* (%)	0 (0.0%)	2 (0.5%)	> .999

*GnRH*, gonadotropin-releasing hormone

### Predictive Factors of Ovarian Response

Multiple regression analysis with backward elimination was performed on a sample of 238 subjects (who had no missing data on potential predictive factors of ovarian response) in the GnRH antagonist group. This model showed, that among the 11 potential predictive factors, the number of retrieved oocytes was significantly predicted by BMI, basal serum FSH and AMH levels, and AFC (Table 4). This model accounted for 26.5% of the variability in ovarian response (*R*^2^ = 0.265, adjusted *R*^2^ = 0.252), and multi-collinearity was diagnosed with the variance inflation factors, tolerance, and condition index, resulting in no evidence of collinearity between independent variables. Among these predictors, basal serum AMH level and AFC were also identified as highly important predictive variables using the random forest method (percentage increase in mean squared error = 17.0% for AMH level and 13.4% for AFC).

**Table 4:** Predictors of the number of retrieved oocytes in the GnRH antagonist group from the multiple regression analysis with backward elimination

Variable	Regression coefficient	Standard error	*P*-value
Intercept	17.1	3.0	< 0.001
Basal serum AMH	0.9	0.2	< 0.001
AFC	0.3	0.1	< 0.001
BMI	−0.4	0.1	< 0.001
Basal serum FSH	−0.3	0.2	0.038

*AFC*, antral follicle count; *AMH*, anti-Müllerian hormone; *BMI*, body mass index; *FSH*, follicle-stimulating hormone; *GnRH*, gonadotropin-releasing hormone

The formula for predicting ovarian response obtained from the final model was as follows: number of oocytes = 17.071 + 0.946 × basal serum AMH (ng/mL) + 0.275 × AFC − 0.363 × BMI (kg/m^2^) − 0.336 × basal serum FSH (mIU/mL).

## Discussion

In infertile female patients enrolled in this multicenter prospective observational study, controlled ovarian stimulation was induced with rhFSH (Follitrope™) using the GnRH agonist or GnRH antagonist protocol at the discretion of the investigator. The mean number of oocytes retrieved was 13.4 in the GnRH agonist group and 13.6 in the GnRH antagonist group. The number of oocytes retrieved is used as a robust surrogate endpoint for healthy live births, which is an important goal of IVF and embryo transfer in infertile women [7, 10]. The number of retrieved oocytes in this study was within the range of 10–15 considered to maximize the live birth rate while reducing the risk of OHSS [7, 13, 14]. As a backing to this affirmation, the clinical pregnancy rate was slightly higher in the subgroup with 10–15 retrieved oocytes than in the other two subgroups (< 10 and > 15 retrieved oocytes). Furthermore, in the subgroup in which more than the appropriate number of oocytes was retrieved (i.e. more than 15), the incidence of OHSS increased significantly, while the clinical pregnancy rate slightly decreased. This supports the finding that the retrieval of more than 15 oocytes significantly increases the risk of OHSS without increasing the rate of live births [13].

The number of retrieved oocytes in this study was within the range of the results of other clinical studies in which controlled ovarian stimulation was induced by the administration of other forms of rhFSH [4, 15, 16]. The results obtained in this study, conducted as an observational study without intervention, were comparable to those of the clinical trials in which rhFSH (Follitrope™) was administered using the GnRH agonist or GnRH antagonist protocol (the mean number of retrieved oocytes varied between 11.8 and 14.9), thus confirming the effectiveness of rhFSH (Follitrope™) in real-world practice [1, 17].

Other clinical outcomes such as fertilization and implantation rates were also within the range of the results of previous studies [1, 15, 16, 18–20]. Furthermore, the clinical pregnancy rate (32.3% in the GnRH agonist group and 39.9% in the GnRH antagonist group) in this study was similar to the clinical pregnancy rate of 41.4% found in a similar observational study in which rhFSH (follitropin alpha) was administered according to the GnRH antagonist protocol in real-world clinical practice [4]. The ongoing pregnancy rate confirmed at least 10 weeks after ovum pick-up (18.3% in the GnRH agonist group and 28.1% in the GnRH antagonist group) was slightly lower than the clinical pregnancy rate. As this was an observational study, no intervention could be performed. It was difficult to accurately identify ongoing pregnancies at up to 10 weeks after ovum pick-up because there were some losses in follow-up cases (due to reasons such as transfer to another obstetric hospital after biochemical pregnancy was confirmed). Accordingly, a phone call follow-up was conducted; however, some subjects (one in the GnRH agonist group and 44 in the GnRH antagonist group) could not be reached. These subjects were not counted as being pregnant at 10 weeks after ovum pick-up (i.e. ongoing pregnancy), resulting in a decrease in the pregnancy rate. In addition, more than 25% of the participants withdrew from the study. Dropouts due to adverse events and other reasons were the most common. Embryo freezing due to the poor subject’s baseline characteristics, other personal patient-related reasons, or the occurrence of adverse events (such as OHSS) led to subject dropout at the discretion of the investigator. Among the subjects from whom the oocytes were retrieved, the subjects who failed to undergo embryo transfer were eight out of 112 in the GnRH agonist group and 50 out of 363 in the GnRH antagonist group. Despite these limitations, this study is meaningful in that it shows the results obtained after administering rhFSH (Follitrope™) in real practice to a large number of infertile patients, unlike clinical trials where rhFSH is usually administered according to predetermined protocols (either the GnRH agonist or GnRH antagonist protocol).

As this is a non-randomized and non-interventional observation study evaluating the clinical outcomes of rhFSH (Follitrope™) in real-world practice (at the investigator’s discretion), there were differences in baseline characteristics and the number of subjects enrolled for each group. Therefore, all of these differences need to be taken into account when interpreting the data obtained from the comparative test between the GnRH agonist and GnRH antagonist groups. For example, the higher AMH level in the GnRH antagonist group affected the clinician’s decision to use this protocol if they considered OHSS risk. This difference reflecting the real-world practice could be the cause of generally similar but different outcomes between the two groups. Even with the imbalances discussed above, clinical outcomes such as biochemical pregnancy rate and clinical pregnancy rate were generally similar between the two groups. Despite the significant difference in the clinical pregnancy rate with a fetal heartbeat, there was no significant difference in the ongoing pregnancy rate which was confirmed at least 10 weeks after ovum pick-up. As emphasized by the uncontrolled observational aspects of this study, the comparative results between the GnRH agonist and antagonist groups that were conducted for exploratory purposes should be interpreted with caution, and it is difficult to conclude that the difference observed in the clinical outcome was due to the effect of a different protocol. The clinical efficacy and safety of rhFSH (Follitrope™) evaluated in the randomized controlled trial using the GnRH agonist protocol were observed in the real-world practice using the same drug with both GnRH agonist and antagonist [1].

It is important to obtain an appropriate number of oocytes that will help achieve optimal clinical outcomes while lowering the risk of OHSS. Predictive factors affecting ovarian response during IVF procedures were identified based on the number of retrieved oocytes used as surrogate markers in infertile female patients undergoing IVF [7, 10]. BMI, basal serum FSH and AMH levels, and AFC were identified (by multiple regression analysis with backward elimination) as predictive factors of the number of retrieved oocytes. Among these predictors, basal serum AMH level and AFC were identified as highly important predictive variables using the random forest method. These results support the findings of other studies which concluded that ovarian response depends on two biomarkers that evaluate ovarian reserve: AFC and serum AMH level [9, 21–24]. Especially, the serum AMH level has a low variability and has been widely used recently to predict ovarian response [9, 25]. The ovarian response is affected by several factors including the patient’s characteristics [26]. Important factors affecting ovarian response could be identified from the available information collected in this study. The regression model in this study accounted for 26.5% of the variability in ovarian response. Identifying these predictors will be a good cornerstone for deriving optimal clinical outcomes that can improve the clinical efficacy (increase pregnancy rates) while lowering the risk of OHSS.

It is worth analyzing the clinical efficacy and safety data from real-world practice, which was not assessed in the phase 3 trial. It is also meaningful to fill the gap by obtaining efficacy and safety data of Follitrope™ using different protocols, as other follitropin products (e.g., Gonal-f) have reported that their data were obtained using different protocols and have reported efficacy of follitropin may vary depending on the manufacturer. This study’s results can be helpful to patients and clinicians using and considering Follitrope™ in their clinical practice.

This study has some limitations. As mentioned above, some subjects withdrew from the study for diverse reasons (inability to undergo fresh embryo transfer, loss to follow-up, etc.), and the use of GnRH agonist or GnRH antagonist was left to the discretion of the investigator; thus, a single controlled ovarian stimulation protocol was not used (multiple agents were used). Therefore, data obtained from each subgroup should be interpreted with caution. Nevertheless, since this study was an observational study without interventions, clinical outcomes using rhFSH could be derived from routine medical practice. We can therefore consider that the results of a heterogeneous population were reflected, and we can expect that rhFSH (Follitrope™) can be used more widely given the evidence of clinical data in real practice. Although ongoing pregnancy data up to 10 weeks after oocyte pick-up were included, the live birth outcome was not included in this study. Many fertility clinics refer their patients for delivery, thus, it is impossible to collect live birth data in this study.

In conclusion, rhFSH (Follitrope™) is safe and effective in inducing controlled ovarian stimulation in infertile women undergoing IVF in real-world practice. Predictors of ovarian response have also been identified. It is expected that the patients’ characteristics identified as predictors (BMI, basal serum FSH and AMH levels, and AFC) in this large-scale observational study can be considered highly related to optimal clinical outcomes.

## Supplementary Materials

ESM 1

## Data Availability

Data analyzed for this study are included in this article.
