# Supplementary material for: Effectiveness and Safety of Recombinant Human Follicle-Stimulating Hormone (Follitrope™) in Inducing Controlled Ovarian Stimulation in Infertile Women in Real-World Practice: a Prospective Cohort Study
Source: Reprod Sci. 2023 Apr 17;30(9):2842–52. doi: 10.1007/s43032-023-01228-6 (PMC10480279; doi:10.1007/s43032-023-01228-6)
Supplement: Supplementary file 1 — ESM 1 [file 43032_2023_1228_MOESM1_ESM.docx]

**Supplementary Table S1 Clinical outcomes according to the number of retrieved oocytes in the GnRH antagonist group (Effectiveness set)**

|  | < 10 oocytes  (N = 117) | 10–15 oocytes  (N = 134) | > 15 oocytes  (N = 112) | *P*-value |
| --- | --- | --- | --- | --- |
| Serum E_2_ level on hCG trigger day (pg/mL) | 2123.8±1297.0 | 2870.6±2378.8 | 3984.3±2451.9 | <.001* |
| Implantation rate (%) | 22.2 (44/198) | 29.7 (70/236) | 24.4 (33/135) | 0.193^†^ |
| Biochemical pregnancy rate (%) | 42.3 (41/97) | 47.5 (56/118) | 45.2 (33/73) | 0.749^†^ |
| Clinical pregnancy rate (%) | 37.1 (36/97) | 41.5 (49/118) | 41.1 (30/73) | 0.784^†^ |
| Clinical pregnancy rate with fetal heartbeat (%) | 30.9 (30/97) | 37.3 (44/118) | 39.7 (29/73) | 0.448^†^ |
| Ongoing pregnancy rate (%) | 23.7 (23/97) | 28.0 (33/118) | 34.2 (25/73) | 0.318^†^ |

Data are presented as mean±standard deviation unless otherwise indicated.

* *P*-values were obtained using the Kruskal-Wallis test.

^†^ *P*-values were obtained using Pearson's chi-square test.

E_2_, estradiol; GnRH, gonadotropin-releasing hormone; hCG, human chorionic gonadotropin.

**Supplementary Table S2 Clinical outcomes according to the agent of GnRH antagonist (Effectiveness set)**

|  | Ganirelix  (N = 214) | Cetrorelix  (N = 147) | *P*-value |
| --- | --- | --- | --- |
| Number of oocytes retrieved | 12.8±6.5 | 14.7±8.6 | 0.158* |
| Serum E_2_ level on hCG trigger day (pg/mL) | 3180.0±2643.4 | 2659.7±1442.4 | 0.473* |
| Fertilization rate (%) | 74.0±22.5 | 78.6±15.6 | 0.207* |
| Implantation rate (%) | 27.2 (88/324) | 24.5% (59/241) | 0.473^†^ |
| Biochemical pregnancy rate (%) | 48.5 (80/165) | 41.3 (50/121) | 0.229^†^ |
| Clinical pregnancy rate (%) | 41.8 (69/165) | 38.0 (46/121) | 0.517^†^ |
| Clinical pregnancy rate with fetal heartbeat (%) | 38.8 (64/165) | 32.2 (39/121) | 0.254^†^ |
| Ongoing pregnancy rate (%) | 33.3 (55/165) | 21.5 (26/121) | 0.028^†^ |

Data are presented as mean±standard deviation unless otherwise indicated.

* *P*-values were obtained using Wilcoxon's rank sum test.

^†^ *P*-values were obtained using Pearson's chi-square test.

E_2_, estradiol; GnRH, gonadotropin-releasing hormone; hCG, human chorionic gonadotropin.
